# Three-dimensional morphological changes of the temporomandibular joint and functional effects after mandibulotomy

**DOI:** 10.1186/s40463-017-0184-4

**Published:** 2017-01-28

**Authors:** Mohammed A. Q. Al-Saleh, Kumaradevan Punithakumar, Manuel Lagravere, Pierre Boulanger, Jacob L. Jaremko, John Wolfaardt, Paul W. Major, Hadi Seikaly

**Affiliations:** 1 Orthodontic Graduate Program, School of Dentistry, University of Alberta, 476 Edmonton Clinic Health Academy (ECHA), Edmonton, Alberta T6G 1C9 Canada; 2 Servier Virtual Cardiac Centre, Mazankowski Alberta Heart Institute and Department of Radiology and Diagnostic Imaging, University of Alberta, Edmonton, Alberta T6G 2B7 Canada; 3 Department of Computing Science, Faculty of Science, University of Alberta, Athabasca Hall, Room 411, Edmonton, Alberta T6G 2E8 Canada; 4 Department of Radiology and Diagnostic Imaging, Faculty of Medicine and Dentistry, University of Alberta, 2A2.41 WC Mackenzie Health Science Center, Edmonton, Alberta T6G 2R7 Canada; 5 Division of Otolaryngology Head and Neck Surgery, Department of Surgery, Faculty of Medicine and Dentistry, University of Alberta, 16940-87 Avenue, Edmonton, Alberta T5R 4H5 Canada

**Keywords:** 3D image analysis, TMJ, MRI, CBCT, Mandibulotomy, TMJ segmentation

## Abstract

**Background:**

The midline and paramedian mandibulotomy are surgical procedures that divide the mandibular bone into two halves and disconnects the condylar heads of the TMJ from each other. This study aimed to prospectively evaluate the temporomandibular joint (TMJ) functional and morphological changes after mandibulotomy using a reconstructed 3D models of the TMJ.

**Methods:**

Sixteen adult patients diagnosed with oral and oropharyngeal tumors with planned surgical mandibulotomy (test group, 9 patients) or transoral (control group, seven patients) treatments were included in the study. MRI and CBCT images were obtained immediately preceeding surgery and 6–8 weeks after surgery. Using the MRI-CBCT registered images, TMJ tissues were segmented at the two occasions by the same operator and 3D models were reconstructed for morphological assessment. Changes across time were measured using the volume overlap and Hausdorff distance of the disc and condyle 3D models. Disc-condyle relationship was measured using point-based and color map analysis. To assess the early functional changes, the Jaw function limitation scale (*JFLS*) and the maximum mouth opening were measured. Two-sample Hotelling T^2^ *t*-test was performed to determine the significance of the morphological and clinical outcomes’ differences between the two groups.

**Results:**

The two-sample Hotelling T^2^ *t*-test showed significant differences (T^2^ (df1,df2) = 0.97 (5,26), *p* <0.01) between the mean values of all outcomes among the 2 groups. The change in disc displacement was significantly different between the two groups (*p* <0.05). However, the condylar displacement was not significantly different between the two groups (*p* =0.3). The average of the *JFLS* score was five times larger after mandibulotomy, and was 2 times larger after transoral surgery (*p* < 0.01). Patients showed decrease in the average of the maximum interincisal mouth opening by 11 mm after mandibulotomy, and by 5.4 mm after transoral surgery.

**Conclusion:**

The quantitative assessment of the TMJ showed minimal changes of the condylar position and variable degrees of articular disc displacement associated with the paramedian split mandibulotomy. As well, limited jaw functions and vertical mouth opening were noticed more in the mandibultomy group compared to the transoral group in 6- weeks after surgery.

## Background

The mandible plays an important role supporting the muscles of mastication in executing the stomatognathic functions, involving speech, chewing and swallowing, and in the cosmetic appearance of the lower third of the face [1]. The midline and paramedian mandibulotomy are surgical procedures that divide the mandibular bone into two halves and disconnects the condylar heads of the TMJ from each other. Midline and paramedian mandibulotomy were first introduced in the eighteenth century to gain access to parapharyngeal tumors and the surgical oncologic value of mandibulotomy has been well established in the literature [2]. The same procedure was introduced to manage chronic TMJ dislocation by rotating the condyles separately in an outward direction [3]. Because of the ability to separately rotate the condyle in the glenoid fossa of the temporal bone with midline split mandibulotomy, it was suggested to improve the TMJ stability and transverse discrepancy in orthognathic mandibular advancement surgeries [4–6].

Squamous cell carcinoma (SCC) of the oropharynx and oral cavity represent approximately 50% of the SCC of the head and neck, which today is the 6th most common malignancy [7, 8]. Mandibulotomy remains a common procedure in the management of SCC of the oropharynx and the oral cavity. Midline and paramedian split mandibulotomy provides the widest and most comfortable access to most regions of the aerodigestive tract.

The reported complications of the mandibulotomy include exposure of metal fixation plate, mal-union or non-union defects, oro-cutaneous fistula, malocclusion, tooth loss or mucogingival tissue loss, and lower lip splitting [9–12]. Also, disturbances of the oral functions can result from the interruption of the mandibular continuity and the inevitable associated condylar head dislocation [13, 14]. Various modifications have been suggested to avoid or reduce the mandibulotomy’s associated post-surgical functional and esthetic morbidities [9–12, 14, 15]. The surgical complications versus the procedure’s benefits have been debated in the literature [9–12]. In contrast to the thoroughly studied esthetic and tissue healing consequences of mandibulotomy, the post-surgical functional and morphological changes of the TMJ have been poorly investigated and reported in the literature [14]. Forces applied to the TMJ during mandibulotomy may injure the TMJ capsule and/or disc. Internal disc derangement alters force dynamics, which stimulate maladaptive responses, potentially resulting in altered osseous contours and jaw dysfunction [16, 17].

The purpose of this study was to prospectively evaluate the morphological and functional changes of the TMJ after midline split mandibulotomy compared to a minimally invasive transoral surgery, using 3D models of the TMJ reconstructed from fused MR-CBCT images, Jaw Function Limitation Scale (JFLS), and maximum mouth opening.

## Methods

### Subject recruitment

All adult patients diagnosed with oral and oropharyngeal malignant tumors with planned surgical mandibulotomy or transoral treatments, at the Division of Otolaryngology Head and Neck Surgery, University of Alberta Hospital, were approached to participate in the study. Exclusion criteria were: history of TMJ trauma, mandibular fracture, jaw pain, TMJ noises, TMJ surgery or chemo-radiotherapy; full dentures, severe systemic co-morbid conditions. Thirty-two subjects met the inclusion criteria and agreed to participate in the study. Patients were divided into 2 groups (*n* = 16) based on the surgery type: 1. *Mandibulotomy surgery* (test-group); 2. *Transoral surgery* (control-group). The patients who agreed to participate in the study were provided with an informed consent clarifying the nature and purpose of the study following the Human Research Ethics Board at the University of Alberta’s policies on research using human subjects (Pro00055827). The obtained images of the participating patients are available at the department of Dentistry, University of Alberta. TMJ assessment was performed in two forms, *imaging assessment* and *clinical assessment* at 2 occasions, 1–2 weeks before surgery (Time 1) and 6–8 weeks after surgery and just before starting any planned adjuvant chemo-radiotherapy (Time 2).

### Imaging protocol

Patients underwent Magnetic Resonance Imaging (MRI) and Cone Beam Computed Tomography (CBCT) for the TMJ with mouth closed and teeth in maximum intercuspation using occlusal bite stents made of polyvinylsiloxane [18].

The CBCT scan was acquired with patient in an upright position and Frankfort plane parallel to the floor. Radiation was collimated to avoid the sensitive structures (thyroid and orbits). Scans were performed using the *Second Generation i*-CAT scanner (Imaging Sciences International, Hatfield, USA) at a medium field of view (FOV) setting, 16 cm × 13 cm, scan time of 26 s, voxel size of 0.25 mm, 120 Kvp and 5 mA. The scan included maxilla, mandible and TMJ condyles.

The MRI scan was performed in a supine position without sedation or intravenous contrast agent administration, using a 1.5 Tesla scanner (Siemens, Munich, Germany) with a multi-channel head array coil. Three MRI sequences were obtained: Mouth-closed oblique sagittal Proton Density-weighted (PD) with a small FOV of 13 cm × 13 cm, a slice thickness of 3 mm (14 slices per TMJ), an inter-slice gap of 0.3 mm, an TE 11 msec and a TR of 1800 msec. Mouth-closed mouth oblique sagittal T2 spoiled gradient echo 3D sequence was obtained with a FOV of 14 cm × 12 cm, a slice thickness of 3 mm, an TE of 95 msec, a TR of 36.3 s and a voxel size of 0.8 × 0.5 × 3 mm^3^. Mouth-open oblique sagittal PD was also obtained, with a small FOV 12 cm × 12 cm, a slice thickness of 3 mm (14 slices per TMJ), an inter-slice gap of 0.3 mm spacing, an TE of 15 msec, a TR of 1800 msec and a voxel size of 0.6 × 0.5 × 3.0 mm^3^.

Pretreatment CBCT images were reviewed to screen for dental pathology prior to cancer treatment.

### Imaging assessment of the morphological changes

**Fig. 1:**
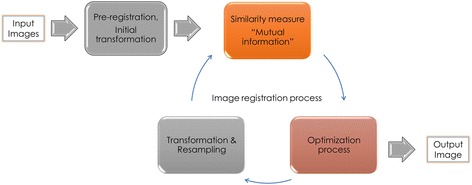
The sequence of different automated image processing steps from the set of two input images to the final fused output image. (*Reproduced from Al-Saleh et al.* [53])

**Fig. 2:**
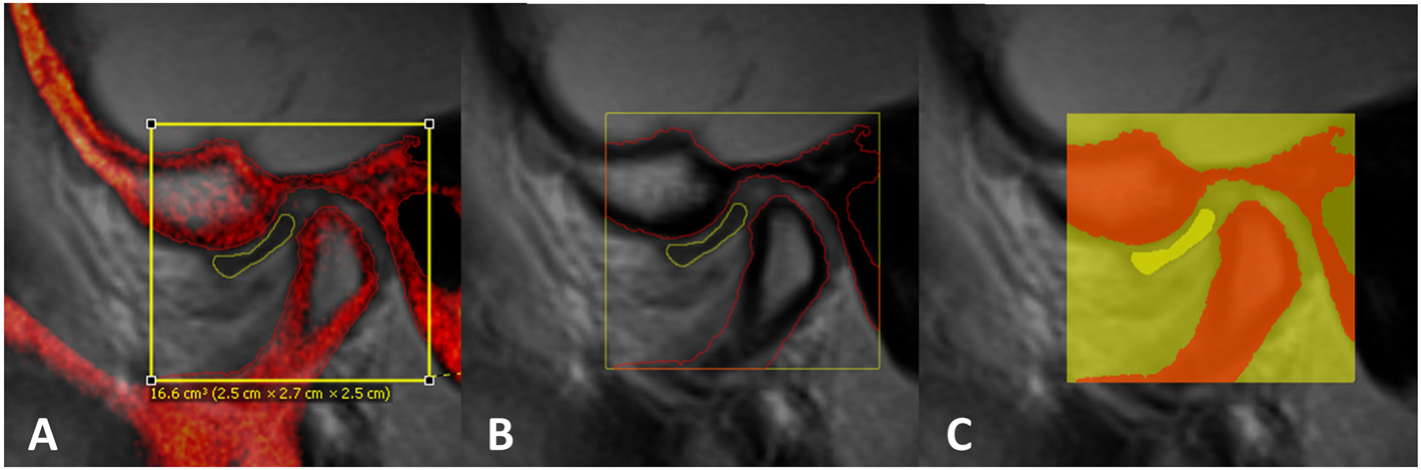
Process of segmentation. **a** Oblique sagittal PD-weighted MRI(*gray*)-CBCT(*red*) registered image showing 3D cropping box (2.5 × 2.7 × 2.5 cm^3^) that was manually drawn to include TMJ articular disc, condylar head, and temporal components. **b** Oblique sagittal PD-weighted MRI only showing the outlined osseous structures (*red*) and articular disc (*yellow*) from the co-registered CBCT image. **c** Same image as B. with highlighted cropped structures to be exported as STL files

The MRI and CBCT images of the TMJs were transferred in the form of Digital Imaging and Communication in Medicine (DICOM) files to Mirada® XD software (Mirada Medical, Oxford, UK) for multi-modality image registration. The multiple MRI sequences of each patient were automatically co-registered with the CBCT image of the same patient. Mutual information rigid image registration algorithm was applied to create common 3D Cartesian coordinate system (x, y, z), for all registered images, which were finally fused into a common display for assessment (Fig. 1). Using the fused image, the gray-value threshold representing the pixel intensity of the condylar head and the glenoid fossa in the CBCT image on each sagittal section was automatically highlighted by Mirada® software. The first author, with post-graduate training in TMD/Orofacial Pain and 5 years dedicated experience working with TMJ MRI and CBCT diagnostic imaging, corrected the outlined structures by adding and erasing as necessary to obtain accurate segmentation of the structures. In the MRI part of the fused image, the articular disc is depicted by low signal intensity in the PD-w and T2-w images. The voxels comprising the articular disc were manually segmented by the first author (Fig. 2). Finally, the segmented tissues were exported in STereoLithography (STL) format and utilized to reconstruct 3D models of the segmented structures using Scan IP software (Simpleware, Exeter, United Kingdom). The segmentation and 3D models reconstruction have been described previously [19].

Changes in condyle, disc, and their relationship, of all joints, from the two occasions were measured and quantified using the 3D model analysis:

1. *Changes in the disc from T1 to T2:*

Disc changes were measured using two parameters:

A. *Dice Similarity Index (DSI):* [20] It measures the degree of overlap between 2 bodies or volumes.

$$ D S I\ \left({M}_{\mathit{\mathsf{1}}},\ {M}_2\right) = 2{M}_{1, 2}/{M}_1 + {M}_2 $$

**Fig. 3:**
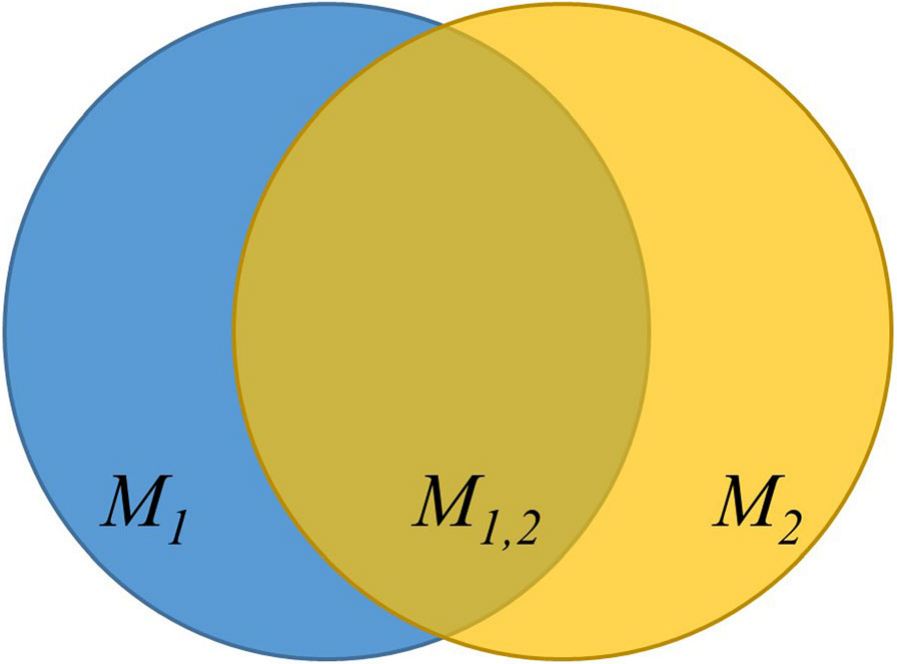
*DSI* measures the overlap between *M*_*1*_ and *M*_*2*_ contours. *DSI* value of 1 indicates full overlap between *M*_*1*_ and *M*_*2*_, DSI value of 0 indicates no overlap between *M*_*1*_ and *M*_*2*_

Where *M*_*1,*_ *M*_*2*_ *and M*_*1,2*_ are the volumes of Time 1 and Time 2 models and the intersection between them respectively (Fig. 3). The *DSI* value is between 0 and 1, where 0 means no overlap between *M*_*1*_ and *M*_*2*_ (full disc displacement) and 1 means perfect overlap (no disc displacement).

**Fig. 4:**
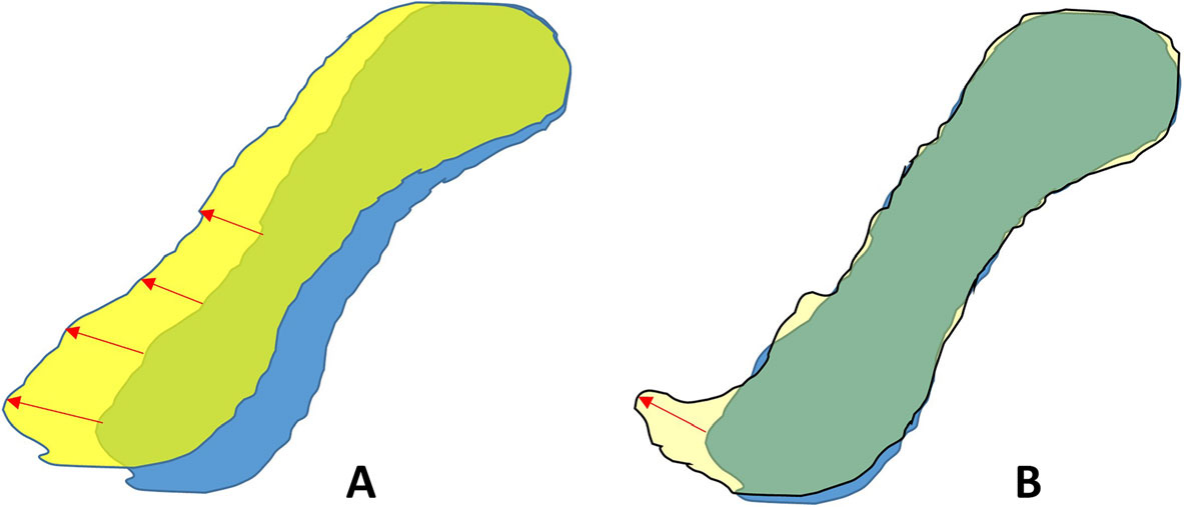
*Red arrows* represents Hausdorff distance. **a** Illustrates two overlapped discs with linear relationship between the *DSI* and the *RMSD* (i.e. low *DSI* value due to displaced disc and high *RMSD*). **b** Illustrates two perfectly overlapped discs with non-linear relationship between the *DSI* and the *RMSD* (i.e. high *DSI* value due to excellent disc overlap, but high *RMSD* value)

B. *The Hausdorff distance:* [21] To quantify the amount of the displacement by measuring the distance between all corresponding surface contour points, in millimeters, at Time 1 and Time 2 (see Fig. 4). The *average perpendicular distance or root mean square distance (RMSD)* was reported as a quantification measure of the *Hausdorff distance*. The relationship between the *DSI* and *RMSD* is not always a linear relationship. Small *RMSD* value does not necessarily indicate excellent overlap (low *DSI*) between two bodies or volumes, but can highlight difference in shape.

  2. *Changes in the condyle from T1 to T2:*

Condylar changes were measured using the same two parameters used in measuring the changes in the dis (i.e. *DSI* and *Hausdorff RMSD*).

3. *Changes in the disc-condyle relationship from T1 to T2:*

To assess changes in *disc-condyle relationship*, point-based analysis was used to produce a color map that quantifies the maximum distance (*MxD*) between the disc and condyle at Time 1 and Time 2. Figures 7, 8, 9 and 10 illustrated the point-based analysis *MxD* in a color mapping scale.

As well, two radiologists with expertise in TMJ imaging subjectively evaluated the disc position and the osseous condition of the subjects’ TMJ before and after surgery. The disc anterior displacement was classified as normal, mild, moderate and severe based on disc position relative to the articulating bony surfaces [18, 22]. The osseous condition of the condyle, articular eminence and glenoid fossa were classified as normal, remodeling (surface flattening and subchondral sclerosis) and degenerative joint disease-DJD (surface erosions, subchondral cyst, osteophyte and joint foreign bodies).

### Clinical assessment of the functional changes

The principal investigator clinically examined all patients at Time 1 and Time 2 and measured the maximum interincisal mouth opening using a millimeter-calibrated Boley gauge. Also, patients were asked to answer 10 questions of the Jaw Function Limitation Scale (*JFLS)* to qualitatively evaluate the mandibular movements’ limitation on their oral activities [23]. The *JFLS* is a numeric scale from 0 = no limitation to 5 = extreme limitation. Patients were asked: ‘How much does your present jaw problem prevent or limit your daily functions?’ Low scores indicated minimum jaw function limitation (Table 1).

**Table 1:** Jaw Function Limitation Scale [23]

How much does your present jaw problem prevent or limit you from….	
1	Talking for a long period of time including telephone conversations.
2	Grinding thin foods.
3	Prolonged chewing during meals.
4	Activity at home, school, and/or work.
5	Clenching teeth when participating in sports (contact teeth together during sports).
6	Opening your mouth widely.
7	Yawning.
8	Brushing your back teeth.
9	Falling asleep.
10	Sleeping through the night.

Score from 0 = No limitation to 5 = Extreme limitation

#### Power, sample size and statistical analysis

The sample size and power were calculated based on published outcomes of the *JFLS*, which was used by Olivo et al. [24] to compare TMJ functions between healthy and TMJ dysfunction group.

When *f* = Sample mean/SD; Sample mean = √(mean – grand mean/number of groups).

According to Portney and Watkins tables, using α of 0.05 and a power of 0.8, a total minimal number of participants that is required to show a difference between the groups, with an effect size of 0.8 and minimum clinically important difference of 5.4 points (~10%), would be seven patients in each arm [24]. Gellrich et al. reported a high dropout rate (50%) in a similar patient group. Therefore, 16 patients were recruited, in each arm, to count for 50% dropout rate [25].

### Statistical analysis

Two-sample Hotelling T^2^ *t*-tests were performed to determine the significance of the morphological and clinical outcomes’ differences between the two surgery types.

The correlation between the imaging and clinical outcomes (*RMSD, DSI*, difference in disc *MxD*, difference in *JFLS* and difference in mouth opening) was investigated. Spearman’s correlation test (-1 ≥ *r* ≥ 1) was performed to compare data that are not normally distributed. The correlation strength was described using the following guide: (0-0.3 = negligible; 0.31-0.5 = low; 0.51-0.7 = moderate; 0.7-0.9 = high; 0.9-1 = very high) [26].

## Results

**Fig. 5:**
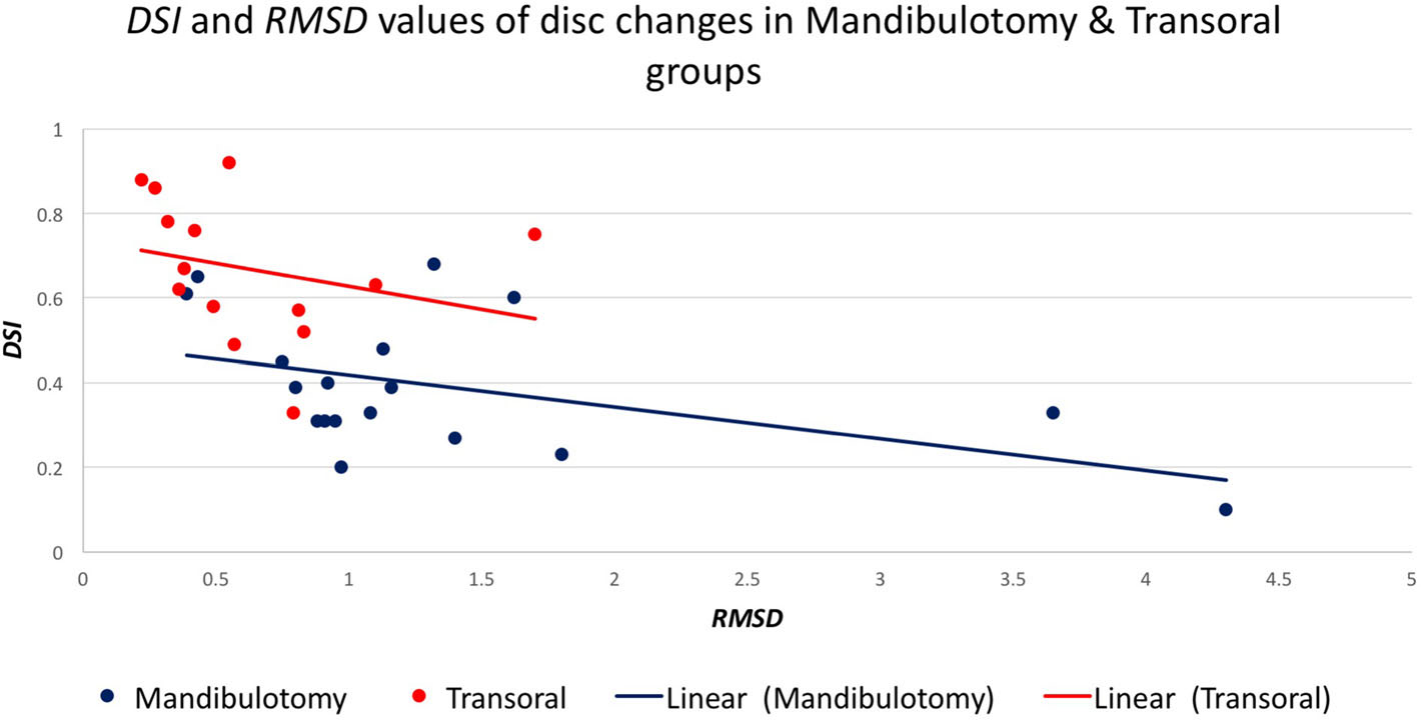
A chart illustrates the values of the *DSI* (Y-axis) and the *RMSD* (X-axis) for disc in mandibulotomy and transoral groups

**Fig. 6:**
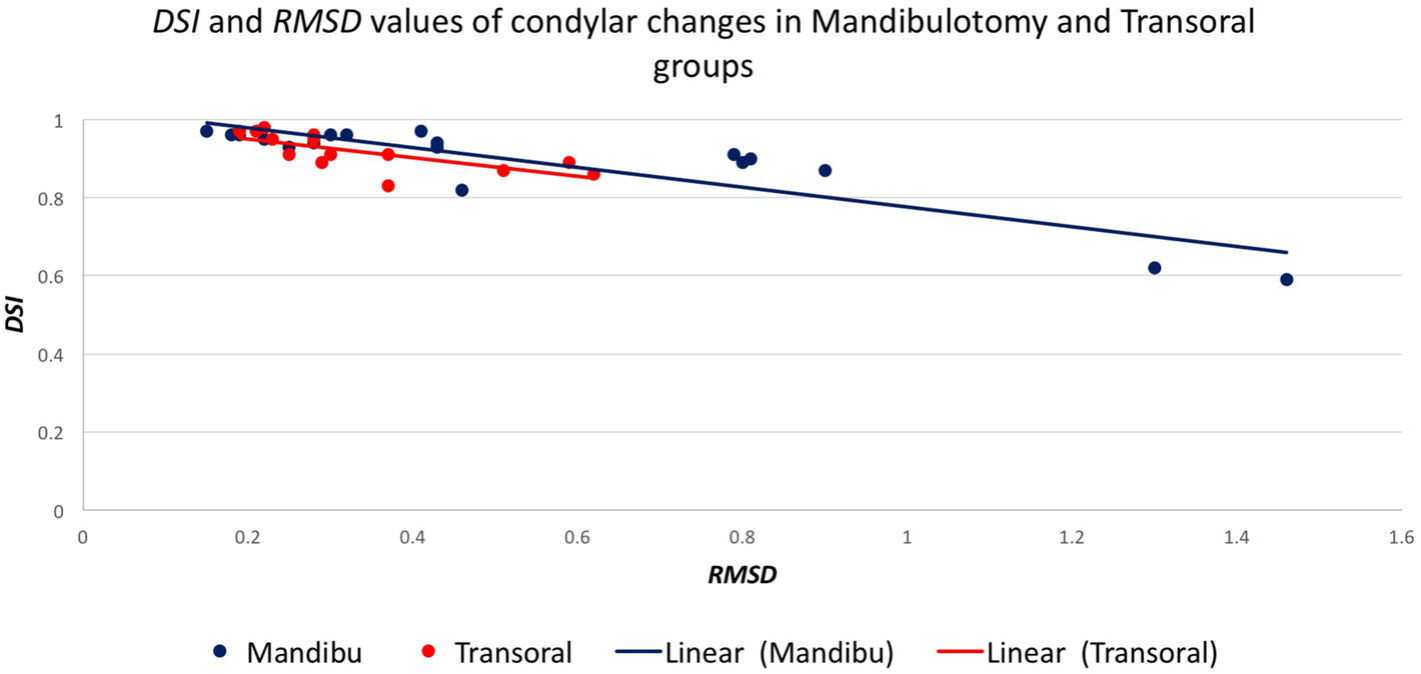
A chart illustrates the values of the *DSI* (Y-axis) and the *RMSD* (X-axis) for condyle in mandibulotomy and transoral groups

**Fig. 7:**
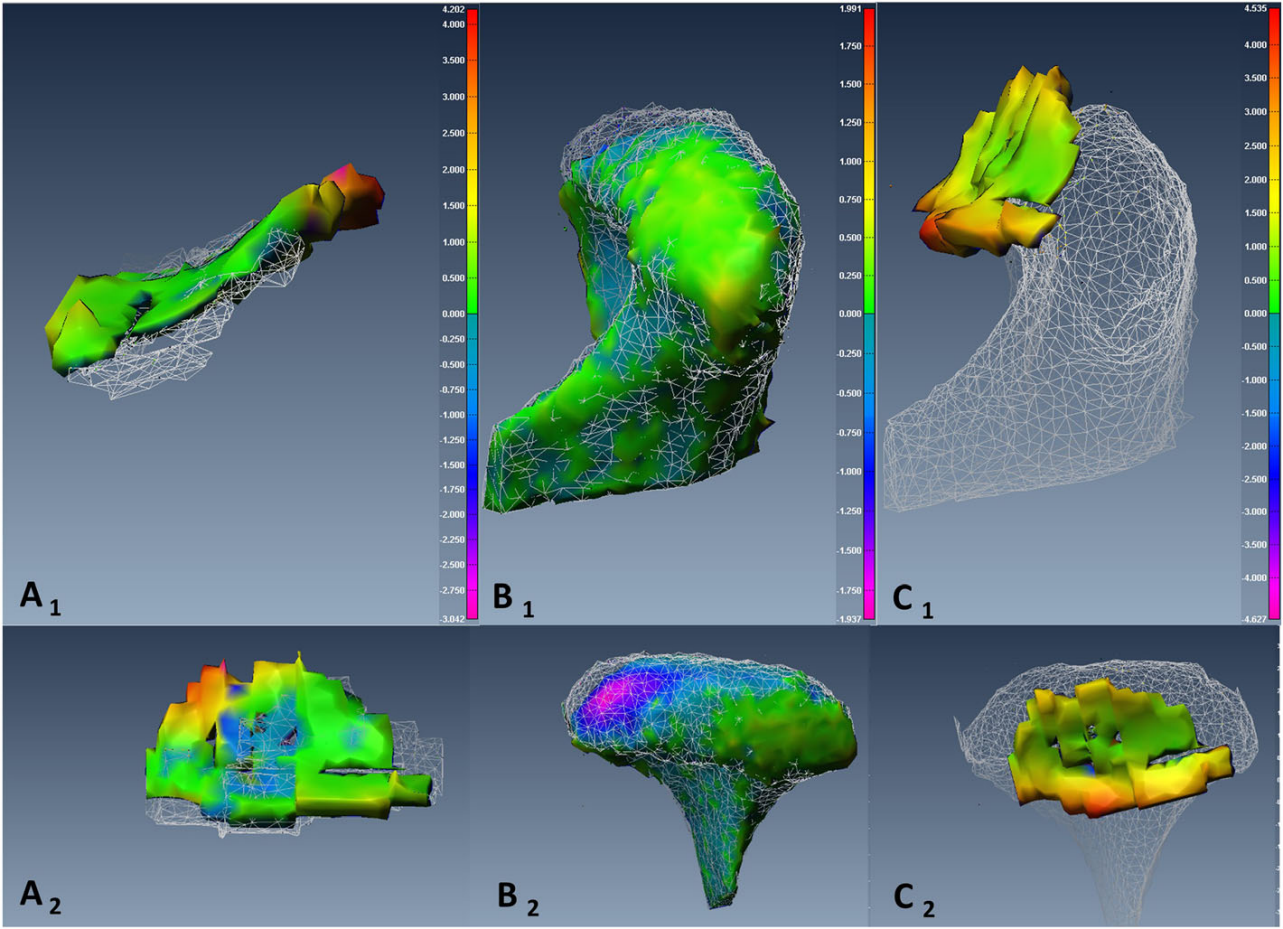
TMJ 3D reconstructed models representative of TMJ from an MRI-CBCT co-registered image of subject number 5 pre- and post-mandibulotomy surgery. The TMJ showed small displacement of the disc and condyle post-surgery compared to pre-surgery. *A*_*1, 2*_: Sagittal and axial views of the same disc illustrate the point-based between pre-operative (white mesh) and post-operative (smooth body) of the disc (*Color code ranges from 4.0 to −3.0 mm*). *B*_*1, 2*_: Sagittal and axial views of the same condyle illustrate the point-based analysis between pre-operative (*white mesh*) and post-operative (smooth body) of the condyle (*Color code ranges from 1.9 to −1.9 mm*). *C*_*1, 2*_: Sagittal and axial views illustrate the point-based analysis of the disc-condyle relationship pre-operatively (*Color map ranged from 4.5 to −4.6 mm*)

**Fig. 8:**
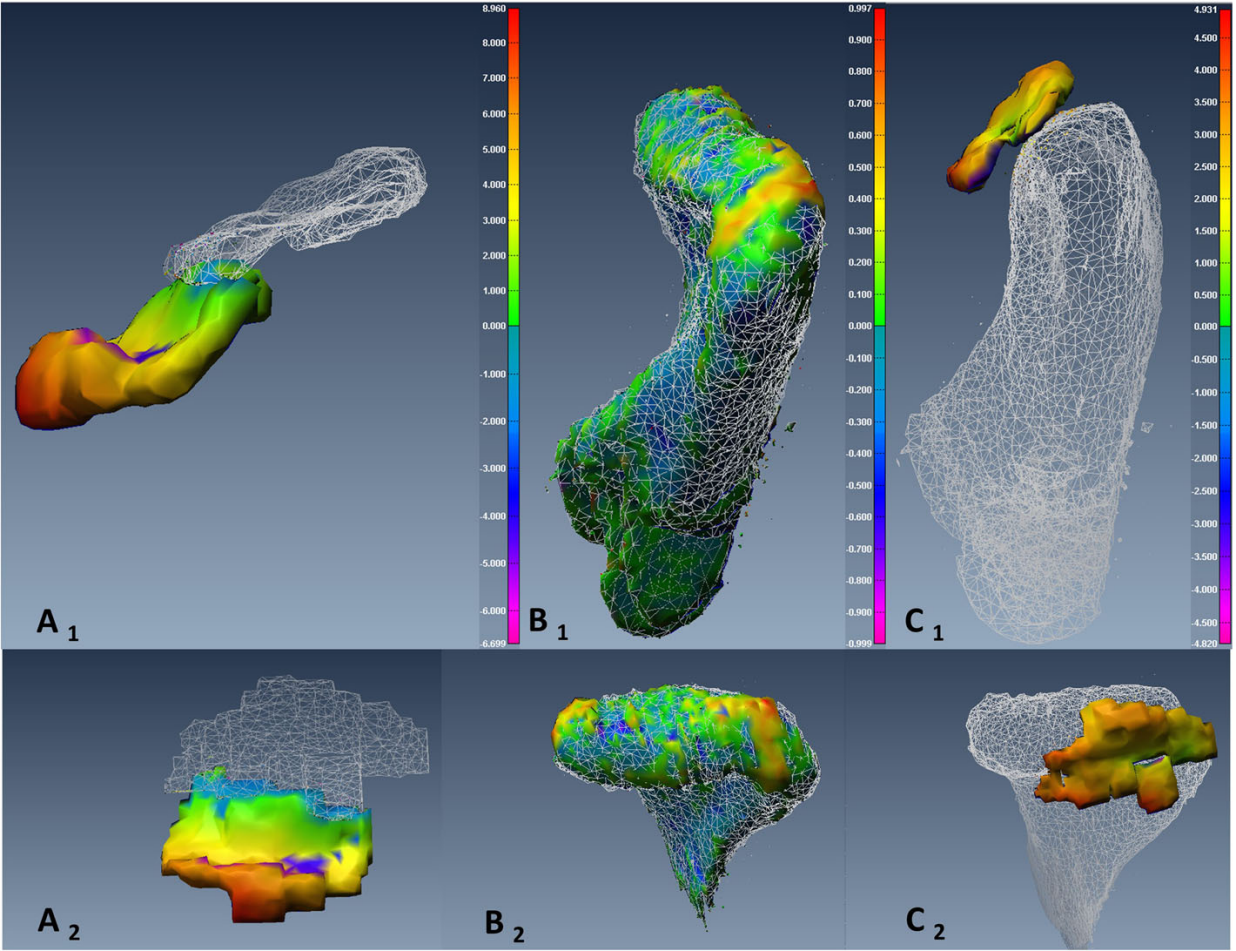
TMJ 3D reconstructed models representative of TMJ from an MRI-CBCT co-registered image of subject number 4 pre- and post-mandibulotomy surgery. The TMJ showed small condylar change and large disc anterior displacement post-surgery compared to pre-surgery. *A*_*1, 2*_: Sagittal and axial views of the same disc illustrate point-based analysis between pre-operative (white mesh) and post-operative (smooth body) of the disc surfaces (*Color code ranges from 9.0 to −6.7 mm*). *B*_*1, 2*_: Sagittal and axial views of the same condyle illustrate point-based analysis between pre-operative (white mesh) and post-operative (smooth body) of the condyle surfaces (*Color code ranges from 1 to −1 mm*). *C*_*1, 2*_: Sagittal and axial views illustrate point-based analysis of the disc-condyle relationship pre-operatively (*Color code ranges from 4.9 to −4.8 mm*)

**Fig. 9:**
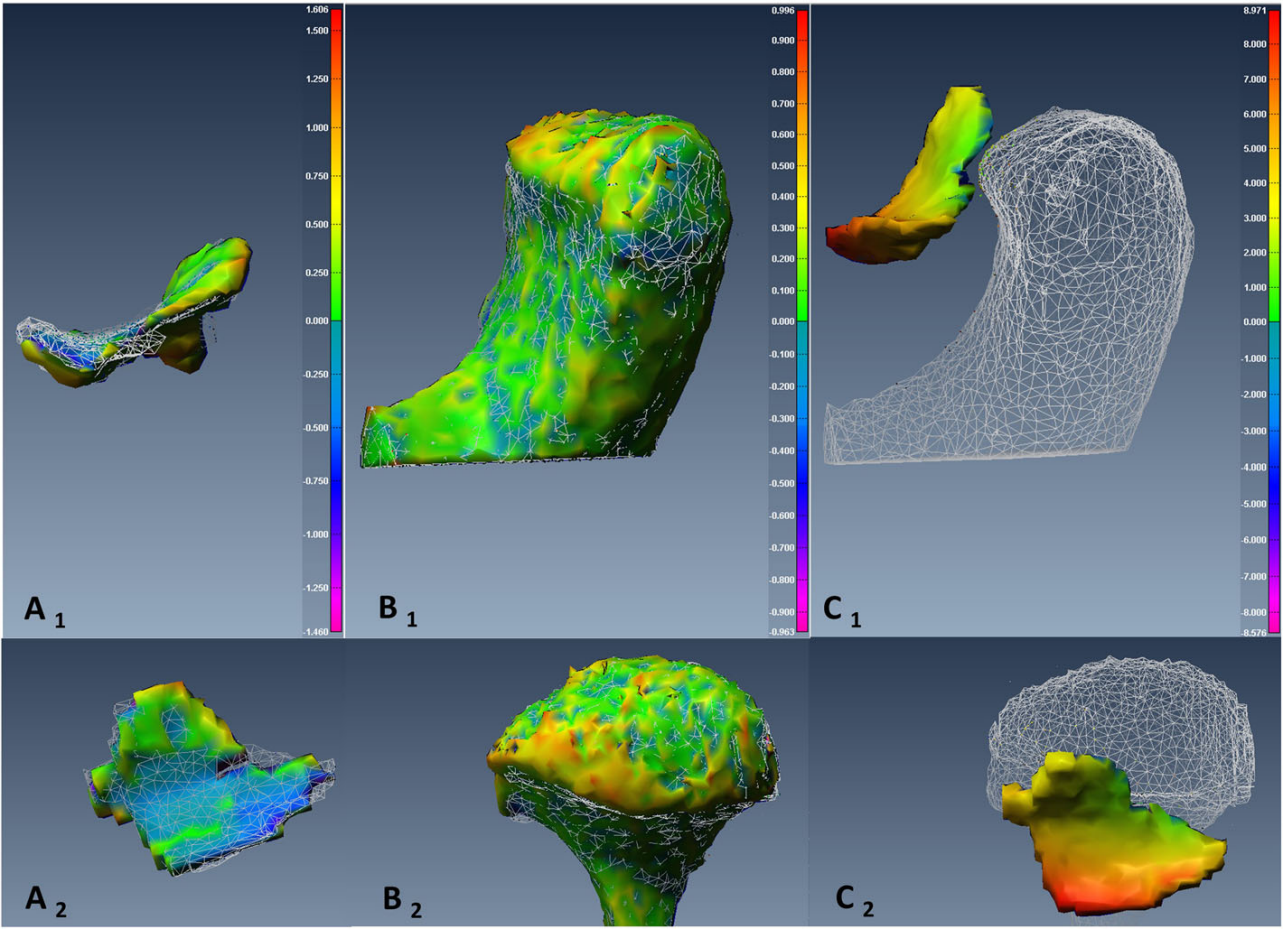
TMJ 3D reconstructed models representative of TMJ from an MRI-CBCT co-registered image of subject number 3 pre- and post-transoral surgery. The TMJ showed small change in disc and condyle positions between pre- and post-surgery. *A*_*1, 2*_: Sagittal and axial views of the same disc illustrate point-based analysis between pre-operative (*white mesh*) and post-operative (smooth body) of the disc surfaces (*Color code ranges from 1.6 to −1.5 mm*). *B*_*1, 2*_: Sagittal and axial views of the same condyle illustrate point-based analysis between pre-operative (*white mesh*) and post-operative (smooth body) of the condyle surfaces (*Color code ranges from 1 to −1 mm*). *C*_*1, 2*_: Sagittal and axial views illustrate point-based analysis of the disc-condyle relationship pre-operatively (*Color code ranges from 8.9 to −8.5 mm*)

**Fig. 10:**
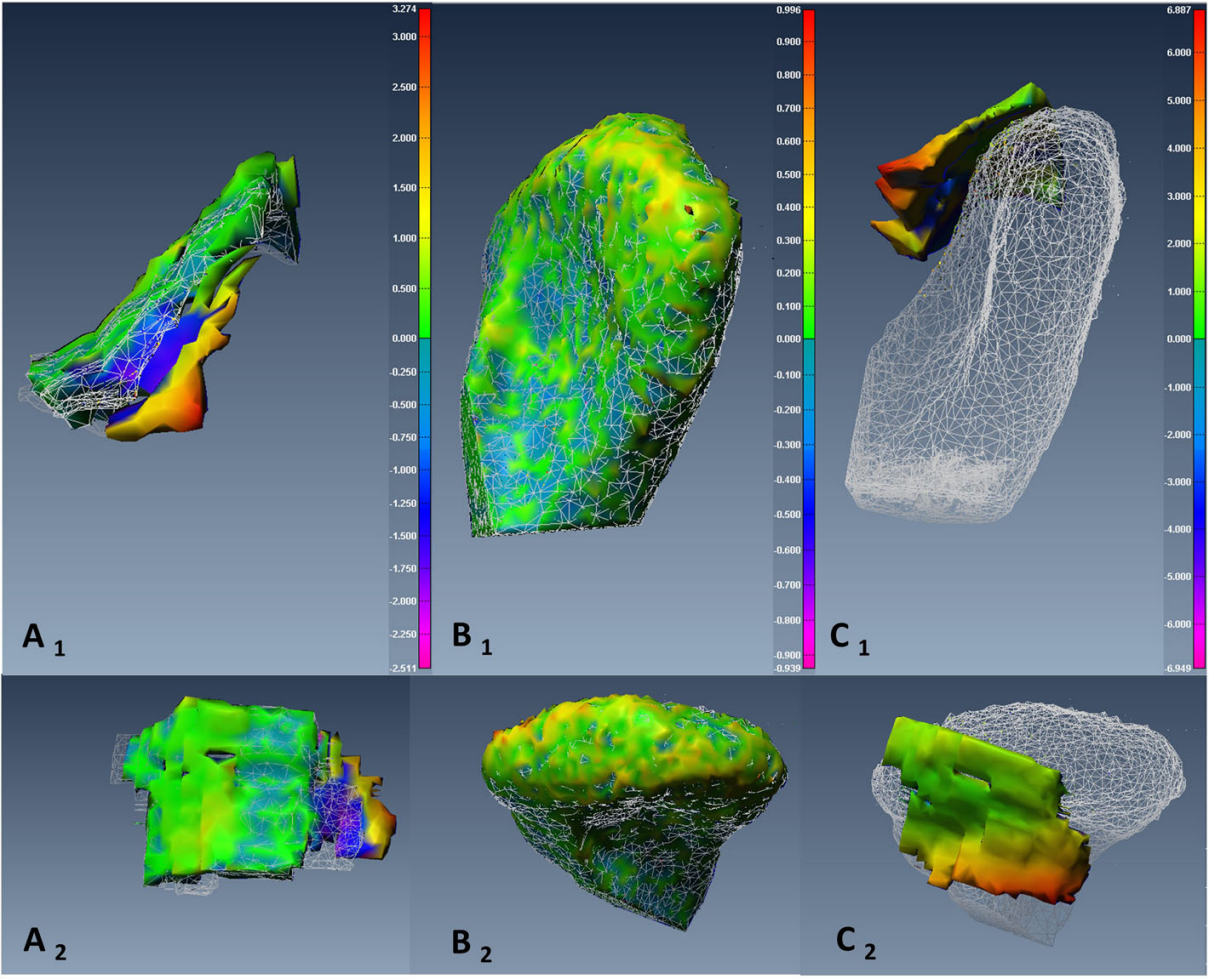
TMJ 3D reconstructed models representative of TMJ from an MRI-CBCT co-registered image of subject number 7 pre- and post-transoral surgery. The TMJ showed small change in disc and condyle positions between pre- and post-surgery. *A*_*1, 2*_: Sagittal and axial views of the same disc illustrate point-based analysis between pre-operative (*white mesh*) and post-operative (smooth body) of the disc surfaces (*Color code ranges from 3.2 to −3.5 mm*). *B*_*1, 2*_: Sagittal and axial views of the same condyle illustrate point-based analysis between pre-operative (white mesh) and post-operative (smooth body) of the condyle surfaces (*Color code ranges from 1 to −1 mm*). *C*_*1, 2*_: Sagittal and axial views illustrate point-based analysis of the disc-condyle relationship pre-operatively (*Color code ranges from 6.9 to −6.9 mm)*

Out of 32 patients, only 16 patients (9 patients from mandibulotomy; 7 patients from transoral) were involved in Time 2 assessments and completed the study. The dropout rate was 43% in mandibulotomy group and 56% in transoral group. Details of the patients’ demographics, tumor type and stage, and treatment type were summarized in Table 2. The changes in disc and condyle as measured by *DSI* and *RMSD* were reported in Tables 3 and 4 and Figs. 5 and 6. As well, the *disc-condyle* relationship, mouth opening and *JFLS* values at Time 1 and Time 2 were reported in Tables 3 and 4. Figures 7, 8, 9 and 10 showed 3D models of 4 representative TMJs from the 2 groups illustrating the *condyle-disc relationship* in color mapping scales.

**Table 2:** Details of the patients’ demographics, tumor type and stage and treatment type

*Age & gender*	*Tumor type, location (Stage)*	*Tumor resection surgery*
50 years Female	SCC, left tongue and tonsils (T4N2M0)	Mandibulotomy
62 years Male	SCC, base of tongue (T3N2M0)	
67 years Male	SCC, base of tongue and right tonsils (T3N2M0)	
60 years Male	SCC, base of Tongue + right tonsils (T3N1M0)	
67 years Male	SCC, left tonsils (T4N2M0)	
64 years Male	SCC, base of tongue (T3N3M0)	
27 years Female	SCC, left lateral tongue (T3N0M0)	
34 years Female	SCC, left tongue (T3N2M0)	
57 years Male	SCC, left tonsil & left tongue (T3N2M0)	
35 years Female	Adenoid cystic carcinoma, palate and U Lip.	Transoral
33 years Male	Adeno carcinoma, left cheek.	
63 years Male	SCC, right lateral tongue, (T4N0M0)	
55 years Female	Papillomatous lesion in the left tonsils.	
54 years Male	SCC, base of tongue and tonsils. (T3N2M0)	
53 years Male	SCC, left tonsil (T1N2M0)	
61 years Male	SCC, right base of tongue (T2N2M0)	

*SCC* squamous cell carcinoma. The TNM Staging System is based on the extent/size of the tumor (T), the extent of spread to the lymph nodes (N), and the presence of metastasis (M) [27]

**Table 3:** The morphological and functional findings of the mandibulotomy group

*Joint #*	*Condyle*		*Disc*		* Disc-condyle relationship (MxD mm)*		*Mouth opening (mm)*		*JFLS*	
	*DSI*	*RMSD*	*DSI*	*RMSD*	*T1*	*T2*	*T1*	*T2*	*T1*	*T2*
*1*	0.82	0.46	0.31	0.91	3.46	5.77	37	29	5	18
*2*	0.93	0.25	0.39	0.8	2.1	4.2				
*3*	0.89	0.8	0.45	0.75	4.9	7.1	52	40	8	22
*4*	0.59	1.46	0.33	3.65	2.43	5.85				
*5*	0.87	0.9	0.27	1.4	2.96	6.5	55	42	0	26
*6*	0.93	0.43	0.2	0.97	2.3	5.1				
*7*	0.62	1.3	0.31	0.95	5.11	7.8	59	43	3	18
*8*	0.91	0.79	0.1	4.3	5.1	1.2				
*9*	0.96	0.32	0.6	1.62	3.1	4.5	54	45	7	19
*10*	0.94	0.28	0.39	1.16	2.46	5.2				
*11*	0.96	0.19	0.33	1.08	1.9	4.3	56	44	2	9
*12*	0.97	0.15	0.48	1.13	2.2	4.1				
*13*	0.96	0.18	0.4	0.92	3.97	5.1	47	36	3	13
*14*	0.95	0.22	0.23	1.8	5.2	2.1				
*15*	0.97	0.41	0.61	0.39	2.31	3.7	49	42	2	10
*16*	0.9	0.81	0.65	0.43	2.1	3.3				
*17*	0.96	0.3	0.31	0.88	4.9	2.2	57	47	0	13
*18*	0.94	0.43	0.68	1.32	4.69	5.7				
*Average*	*0.9*	*0.5 mm*	*0.4*	*1.4 mm*	*3.4 mm*	*4.7 mm*	*51.7 mm*	*40.8 mm*	*3.3*	*16.4*
*Average of difference between T1& T2*					*1.3 mm*		*10.9*		*13.1*	

*DSI* dice score index, *RMSD* root mean squared distance, *JFLS* jaw function limitation scale

**Table 4:** The morphological and functional findings of the transoral group

*Joint #*	*Condyle*		*Disc*		*Disc-condyle relationship (MxD mm)*		*Mouth opening*		*JFLS*	
	*DSI*	*RMSD*	*DSI*	*RMSD*	*T1*	*T2*	*T1*	*T2*	*T1*	*T2*
*1*	0.91	0.3	0.62	0.36	2.5	3.6	53	48	0	2
*2*	0.95	0.28	0.58	0.49	2.5	1.5				
*3*	0.89	0.59	0.75	1.7	2.9	3.8	59	53	4	3
*4*	0.91	0.37	0.63	1.1	4	5.3				
*5*	0.96	0.28	0.76	0.42	5.1	6.05	56	49	5	7
*6*	0.86	0.62	0.57	0.81	3.3	4.3				
*7*	0.95	0.23	0.92	0.55	1.59	2.01	49	48	4	8
*8*	0.91	0.25	0.33	0.79	2.11	3.8				
*9*	0.97	0.21	0.86	0.27	1.88	2.5	53	49	0	4
*10*	0.83	0.37	0.49	0.57	1.47	3				
*11*	0.97	0.19	0.88	0.22	2.71	3.5	57	48	0	3
*12*	0.89	0.29	0.78	0.32	2.52	3				
*13*	0.87	0.51	0.52	0.83	3.04	3.8	55	49	2	6
*14*	0.98	0.22	0.67	0.38	2.16	0.3				
*Average*	*0.9*	*0.3 mm*	*0.7*	*0.6 mm*	*2.7 mm*	*3.3 mm*	*54.5 mm*	*49.1 mm*	*2.1*	*4.7*
*Average of difference between T1& T2*					*0.6 mm*		*5.4 mm*		*2.6*	

*DSI* dice score index, *RMSD* root mean squared distance, *JFLS* jaw function limitation scale

The two-sample Hotelling T^2^ *t*-test showed significant differences (T^2^ (df1,df2) = 0.97 (5,26), *p* <0.01) between the mean values of all outcomes among the 2 groups. Pairwise comparisons tests showed significant differences among all outcomes (*p* < 0.01) except for two outcomes, the condyle’s *RMSD* and *DSI*. Mean difference and confidence interval of all outcomes were reported in Table 5.

**Table 5:** The effect of the surgery type on the imaging and the clinical outcomes

Outcomes		Mean difference	Std. Error	Significance	95% Confidence interval for difference	
					Lower bound	Upper bound
*Condyle*	*DSI*	0.03	0.03	0.25	−0.10^a^	0.02
	*RMSD (mm)*	0.21	0.10	0.05	0.00	0.43
*Disc*	*DSI*	−0.26	0.05	<0.01	−0.38^a^	−0.14^a^
	*RMSD (mm)*	0.70	0.28	0.02	0.11	1.29
*Disc-condyle relationship (MxD mm)*		1.25	0.25	<0.01	0.73	1.78
*Mouth opening (mm)*		5.00	0.98	<0.01	2.99	7.00
*JFLS*		9.00	1.69	<0.01	5.54	12.45

Mean differences were of the outcomes were evaluated a two-sample Hotelling T^2^ *t*-test. Pairwise comparisons between the outcomes were as follows: *Significance = p < 0.05*

^a^= Transoral surgery values were larger than the mandibulotomy surgery values, hence the negative sign

The average of the maximum mouth opening in the mandibulotomy group before surgery was 51.7 mm and after surgery was 40 mm. For the transoral group, the average of the maximum mouth opening before surgery was 54.5 mm, and after surgery was 49.1 mm. The average of the *JFLS* score in the mandibulotomy group before surgery was 3.3 and after surgery was 16.4. For the transoral group, the average of the *JFLS* score before surgery was 2.1, and after surgery was 4.7.

Spearman’s correlation showed significant and high correlations when:

- The condyle’s *DSI* decreased, the *RMSD* increased (*r*_=_ −0.77, *p* <0.05).
- The disc *DSI* decreased, the *MxD* increased (*r*_=_ −0.88, *p* <0.05), and *JFLS* increased (*r*_=_ 0.76, *p* <0.05).
- The *JFLS* increased, the mouth opening limitation increased (*r*_=_ 0.74, *p* <0.05).

Spearman’s correlation showed significant and moderate correlations when:

- The disc *RMSD* increased, the *MxD* increased (*r*_=_ 0.61, *p* <0.05).
- The disc *DSI* decreased, the mouth opening limitation increased (*r*_=_ 0.57*, p <0.05*).

Table 6 illustrates the pairwise correlations between all outcomes.

**Table 6:** Spearman’s (*r*) correlation between the different outcomes of both groups

		*Condyle*		*Disc*		*Disc-condyle relationship (MxD)*	*Mouth opening*
		*DSI*	*RMSD*	*DSI*	*RMSD*		
*Condyle*	*DSI*						
	*RMSD*	−0.77^*^					
*Disc*	*DSI*	0.31	−0.26				
	*RMSD*	−0.26	0.31	−0.59^*^			
*Disc-condyle relationship (MxD)*		−0.18	0.26	−0.88^*^	0.61^*^		
*Mouth opening*		−0.09	0.25	−0.61^*^	0.57^*^	0.67^*^	
*JFLS*		−0.18	0.37^*^	−0.70^*^	0.49^*^	0.76^*^	0.74^*^

(* = *p* <0.05). 0-0.3 = negligible; 0.31-0.5 = low; 0.51-0.7 = moderate; 0.7-0.9 = high; 0.9-1 = very high. P.S. negative value indicates negative correlation

In the subjective evaluation of the disc and the osseous structures for the 2 groups the findings were as follows:

- *Before mandibulotomy*: Normal disc = 4 joints; Mild disc displacement = 4 joints; Moderate disc displacement = 4 joints; Severe disc displacement = 6 joints; Normal osseous condition = 3 joints; Osseous surface remodeling = 6 joints; DJD = 9 joints.
- *After mandibulotomy*: 2 joints progressed from mild & moderate to severe disc position, and no changed in the osseous condition was noticed.
- *Before transoral*: Normal disc = 5 joints; Mild disc displacement = 5 joints; Moderate disc displacement = 3 joints; Severe disc displacement = 1 joints; Normal osseous condition = 2 joints; Osseous surface remodeling = 7 joints; DJD = 5 joints.
- *After transoral*: neither disc position nor osseous conditions changed after transoral surgery.

## Discussion

The matter of whether the midline or paramedian mandibulotomy negatively impacts the oral functions has been a cause of controversy in the literature [9–12, 14]. In the last decade, several surgical techniques and options have been introduced in the area of head and neck and craniofacial surgery to avoid the potential TMJ trismus or functional limitation. However, morphological and clinical changes of the TMJ due to mandibulotomy have not been adequately investigated in the literature [14]. A well-designed experimental study, using valid assessment tools and cohort subjects, was recommended to determine the effect of the mandibulotomy on the TMJ and lead to better understanding of the resultant changes [14].

Adjuvant chemo-radiotherapy was reported to delay healing capacity and restrict the mouth movement [14, 27–31]. In present study, 6–8 weeks follow up appointment just before starting the chemo-radiotherapy was selected to avoid the radiation effect on the TMJ tissues and functions. The follow up period may have not been long-enough for patients to completely heal after surgery, however, the long-term evaluation was outside the purpose of the present study.

The high dropout rate (43 and 56%) in the present study was similar to another study in the literature [25]. Sixteen patients from both groups were not involved in the follow-up appointment for different reasons: (1 died after surgery, 2 had mandibulectomy surgery instead of mandibulotomy, 1 became edentulous and 12 withdrew from the study due to inconvenience). It’s our belief that the intervention-independent reasoning and the almost similar dropout rates in both groups had minimized the resultant bias on the study’s findings. Despite the high dropout rate, the sample size was still at the required level in the transoral group (*n* = 7 patient/group) or slightly higher in the mandibulotomy group (*n* = 7 patient/group).

### Morphological changes

Despite multiple studies in the literature discussing complications and functional outcomes after mandibulotomy and transoral surgeries, none has deeply investigated the morphology changes of the TMJ [25, 32–36]. In the present study, the condylar head and articular disc changes, and their relationship were quantitatively evaluated using reconstructed 3D models representative of TMJ from MRI-CBCT registered images. The MRI-CBT registration process used was recognized as an accurate technique [37], and was reliable when evaluating the TMJ internal disc derangement [18].

The changes of the articular disc and condylar head in 3D space relative to the pre-surgical position were measured independently using two different, yet complementary, parameters. The *DSI* reflected the disturbance of the overall body displacement in a Likert-type scale (score from 0 to 1). However, the amount of the displacement at any direction was measured using the *RMSD*. The articular disc *DSI* and *RMSD* values were more variable than the condyle values and their relationship were not absolutely linear in both groups (Fig. 5) and with only moderate correlation (*r* = −0.59). Figure 5 illustrated a higher range of the disc displacement in the mandibulotomy group compared to the transoral group. The change in disc displacement was significantly different between the two groups, with mean *DSI* difference of 0.25 ± 0.5 (*p* <0.01, *CI* = [−0.38– − 0.14]), and mean *RMSD* difference of 0.7 ± 0.28 mm (*p* =0.02, *CI* = [0.1–1.2]). Two discs (joints no. 4 and 8) in the mandibulotomy group showed maximum displacement with low *DSI* values (Joint no. 4 *DSI* =0.33; *RMSD* = 3.6 mm), (Joint no. 8 *DSI* =0.1; *RMSD* = 4.3 mm). The disc changes between the two groups remained significantly different even when joints no. 4 and 8 were removed and data were re-analyzed (mean *DSI* difference of 0.23 ± 0.4 (*p* <0.01, *CI* = [−0.38– − 0.10]), and mean *RMSD* difference of 0.51 ± 0.18 mm (*p* =0.01, *CI* = [0.1–1.01]). The fact that these two joints were severely anteriorly displaced disc before surgery could have substantially influenced the surgical effect on them after surgery (Fig. 8 illustrated the change in joint no. 4 in 3D model). The condylar head changes showed linear relationship between the *DSI* and *RMSD* values in both groups (Fig. 6), and showed very high correlation (*r* = –0.77). Figure 6 illustrated a higher range of *RMSD* but a small range of the *DSI* indicating limited displacement in of condyle in both groups. the disc displacement in the mandibulotomy group compared to the transoral group. The change in condylar displacement was not significantly different between the two groups, with mean *DSI* difference of 0.03 ± 0.03 mm (*p* =0.3, *CI* = [−0.1–0.02]), and mean *RMSD* difference of 0.21 ± 0.1 mm (*p* = 0.05, *CI* = [0–0.43]). Two condyles (joints no. 4 and 7) in the mandibulotomy group showed maximum displacement values with moderate *DSI* values (Joint no. 4 *DSI* =0.62; *RMSD* = 1.3 mm), (Joint no. 7 *DSI* =0.59; *RMSD* = 1.4 mm). On another note, the point-based analysis of the *disc-condyle relationship* is an accumulative result of the displacement amount of the disc and condyle. The mean difference of the maximum distance (*MxD*) that measured the *disc-condyle relationship* was found to be significantly different between the two groups (*MxD* = 1.25 ± 0.25 mm, *p* <0.01, *CI* = [0.73–1.78]).

The observed larger change in articular disc compared to the condyle can be attributed to many factors related to the nature of the articular disc anatomy, surgical procedures and the 3D segmentation errors. The articular disc ligaments are not elastic and upon stretching they irreversibly elongate [38–42]. Even routine dental procedure or mild trauma can, sometimes, cause an internal disc derangement, which alters force dynamics and potentially result in long-term consequences [38–42]. The severe stretching action of the mandible halves for long hours during mandibulotomy surgery likely resulted in more accentuated disc displacement compared to the transoral surgery group. Moreover, the manual 3D segmentation of the articular disc was found to have a higher marginal error (0.3 ± 0.1 mm) than the semi-automatic condylar 3D segmentation (0.1 ± 0.1 mm) [19]. The successful reunion of the two halves of the mandible using a reliable surgical template and internal rigid fixation could be another factor of the minimal change of the condylar head. The clinical significance of the condylar position is controversial in the current literature [43]. The condylar position was quite variable in the mandibulotomy group, however, the long-term consequences of the change in condylar position remains unknown. As well, the relatively short follow-up period would likely be insufficient to see change in bone morphology due to osseous degeneration. Harris and Heaney reported that a decrease of 30–50% of the skeletal mass is required in order to detect erosive lesions in the radiograph [44]. The gradual demineralization of the bone matrix, however, is a slow process that takes many weeks in humans depends on many factors including age, trauma, dysfunctions and hormonal disturbance [44].

### Clinical changes

The main goal of any cancer surgery is complete excision of the lesion with a clear margin, however, maintaining oral functions to the best possible degree is another important goal. One of the major shortcomings in the published oral and oropharyngeal cancer studies is insufficient description of the clinical examination methods and criteria. Mandibulotomy was suggested to play a causative role in reducing vertical mouth opening and jaw dysfunction [14, 15]. The majority of the literature evaluated oral and oropharyngeal cancer treatment impact on quality of life (QoL), which is a common generic head and neck QoL measure that is not sensitive to oral functions impairment [45, 46].

The *JFLS* is a valid and reliable organ-specific scale that measures the oral and TMJ dysfunctions and the patients’ perception of the social impact on their well-being [23, 47, 48]. In the present study, the average of the *JFLS* score in the mandibulotomy group was 16.4 (almost 5 times larger after surgery), whereas, the average of the *JFLS* score in the transoral group was 4.7 (2 times larger after surgery). The *JFLS’s* mean difference between the two surgery groups was 9 ± 1.69 mm (*p* <0.01, *CI* = [5.5–12.4]). The severity of the TMJ dysfunction in a typical TMD patient was reported to range between 21 and 28 points of the *JFLS* scoring system, and the difference between the healthy group and TMD patients was reported to be 11 points [23, 24]. The highest impairment scores after mandibulotomy were mainly given to three questions: 1. “*Talking for long period of time*”; 2. “*Prolonged chewing*”; and 3. “*Activity at home/work*”. It is possible that with more healing time, these functional limitations may resolve. The high *JFLS* scoreswere highly correlated to the differences in the disc *DSI* value (*r* = -0.70), *disc-condyle relationship* (*r* =0.76), and maximum mouth opening (*r* =0.74). However, this correlation cannot assume cause-effect relationship between the disc displacement and post-operative TMJ dysfunction. TMJ functional changes following mandibulotomy procedure have been reported in multiple studies in the literature. Christopoulos et al. reported long-term (1–10 years) functional performance, and compared mandibulotomy patients versus mandibulectomy patients [35]. Ninety-seven percent of the mandibulectomy patients reported more dysphagia and having soft diets versus 43% of mandibulotomy patients. Riddle et al. reported 1 year post-operative symptoms of local pain and discomfort in mandibulotomy patients using yes or no answers [33]. Six percent reported remaining pain at the midline split site, 32% reported TMJ pain with chewing or speaking, 41% had tenderness or discomfort at the temporalis or masseter muscles associated with TMJ movements. Lee et al. used self-reporting questionnaire to asses swallowing dysfunction in 1 year after transoral-robotic versus transoral/transmandibular surgeries [36]. There was a significant difference in the recovery of full swallowing ability in the three groups of patients who underwent transoral-robotic, transoral and mandibulotomy on average of 6.5 ± 4, 7 ± 8 and 16.7 ± 5 days respectively. Gellrich et al. surveyed 1650 patients who underwent different types of surgical and chemo-radiotherapy treatments for oral SCC tumors [25]. The authors found that the highest impairment reported was in chewing, swallowing and tongue mobility 6 months after surgery in all patients. Likely, the post-operative dysfunction is more related to the amount of the resected oral tissues [25, 49].

Tenderness provoked by TMJ movement correlates to jaw dysfunction [19, 50], Measuring jaw movement capacity in millimeters, especially the vertical movement, is sensitive to over time change and has excellent reliability to determine the severity of limitation of jaw movement [51, 52]. The mandibulotomy group patients showed decrease in the average of the maximum interincisal mouth opening after surgery of about 11 mm. However, 16 out of 18 patients in mandibulotomy group were able to open more than 40 mm, which is considered an acceptable vertical range of movement after a relatively short period of surgical recovery [42]. The transoral group patients showed a slight decrease in the average of the maximum interincisal mouth opening after surgery (~5.4 mm) and all of them were able to open about 50 mm. The mouth opening mean difference between the two groups was 5 ± 0.9 mm (*p* <0.01, *CI* = [2.9–7.0]). Although no direct influence of the joints with severe disc displacement (joints no. 4 and 8) on vertical mouth opening was noticed, the mean difference of the maximum mouth opening was moderately correlated to the change in disc displacement (*RMSD*, *r* = 0.57), morphology (*DSI*, *r* = -0.61) and *disc-condyle* relationship (*r* = 0.67). Christopoulos et al. found no significant difference in mouth opening between mandibulotomy patients (~50 mm) and mandibulectomy patients (~40 mm) [35]. Riddle et al. found that 30% of 93 mandibulotomy patients reported reductions in vertical mouth opening with post-operative average of 41 mm [33]. Overall, limitation in mandibular movement in both vertical mouth opening and lateral movements after mandibulectomy seemed to be attributed to the scarring and prolonged muscle immobility [15, 49]. In some cases, the decrease in mouth opening and movement limitation is likely attributed to the simultaneous soft tissue resection such as pterygoid muscles, adjuvant chemo-radiotherapy and/or attendant reconstruction.

The findings of the present study confirmed the substantial TMJ changes associated with the mandibulotomy when compared to transoral surgery. The associated morphological changes emphasized the minimal condylar changes in both groups, but higher disc displacement in mandibulotomy group compared to transoral group. These changes may be partially responsible for the functional limitation after mandibulotomy and TMJ dysfunction [41, 42]. The slow recovery in the mandibulotomy group could, also, be attributed to the injury of the floor of mouth muscles, constrictor muscle, and pharyngeal nerve plexus, which were minimally injured with the transoral surgery [36]. The 3D reconstructed models from the MRI-CBCT registered images reflected a clear picture of the morphological changes of the TMJ after mandibulotomy and transoral surgeries. To the authors best knowledge, no study has investigated the morphological changes of the TMJ tissues in a similar surgical intervention or patient population. The lack of similar studies made it difficult to compare the present study findings with other studies in the literature. The reported morphological changes provided an important source of information in the field of oral and oropharyngeal surgical management field.

CBCT and MRI imaging provides useful diagnostic information regarding TMJ morphology which can be used to direct treatment to restore jaw function.

This study had several limitations. The follow-up period was short and another study can be attempted to evaluate the long-term effects on the same cohort. Although the patients of both groups were matched in age and gender, the tumor type, size and extension were not completely matched, which may have been a source of bias when the outcomes of the both groups were compared. Also, exploring the morphological changes of the TMJ after the chemo-radiotherapy can be useful in understanding the associated morphological changes to the resulted functional limitations of the TMJ.

## Conclusions

The quantitative assessment of the TMJ using the 3D reconstructed models of MRI-CBCT registered images, showed minimal changes of the condylar position and variable degrees of articular disc displacement associated with the paramedian mandibulotomy. As well, limited jaw functions and vertical mouth opening were noticed more in the mandibultomy group compared to the transoral group in 6- weeks after surgery. A future study with long-term evaluation is advised to detect potential long-term morphological and functional changes of the TMJ.
